# Novel Biomass-Based Polymeric Dyes: Preparation and Performance Assessment in the Dyeing of Biomass-Derived Aldehyde-Tanned Leather

**DOI:** 10.3390/polym15102300

**Published:** 2023-05-13

**Authors:** Wei Ding, Yinuo Zhang, Shuolin Li, Javier Remón, Kanglei Wang, Lihong Bao, Xiaoyan Pang

**Affiliations:** 1 China Leather and Footwear Research Institute Co., Ltd., Beijing 100015, China; 18811756311@163.com (S.L.); wkljy1226@163.com (K.W.); 2 School of Materials Design and Engineering, Beijing Institute of Fashion Technology, Beijing 100029, China; 19801328393@163.com (Y.Z.); clybln@bift.edu.cn (L.B.); 3 Thermochemical Processes Group, Aragón Institute for Engineering Research (I3A), University of Zaragoza, 50018 Zaragoza, Spain; jrn@unizar.es

**Keywords:** dialdehyde starch, reactive red dye, biomass-derived aldehyde, leather tanning, dyeing capabilities, physical properties

## Abstract

High-performance chrome-free leather production is currently one of the most concerning needs to warrant the sustainable development of the leather industry due to the serious chrome pollution. Driven by these research challenges, this work explores using biobased polymeric dyes (BPDs) based on dialdehyde starch and reactive small-molecule dye (reactive red 180, RD-180) as novel dyeing agents for leather tanned using a chrome-free, biomass-derived aldehyde tanning agent (BAT). FTIR, ^1^H NMR, XPS, and UV-visible spectrometry analyses indicated that a Schiff base structure was generated between the aldehyde group of dialdehyde starch (DST) and the amino group of RD-180, resulting in the successful load of RD-180 on DST to produce BPD. The BPD could first penetrate the BAT-tanned leather efficiently and then be deposited on the leather matrix, thus exhibiting a high uptake ratio. Compared with the crust leathers prepared using a conventional anionic dye (CAD), dyeing, and RD-180 dyeing, the BPD-dyed crust leather not only had better coloring uniformity and fastness but it also showed a higher tensile strength, elongation at break, and fullness. These data suggest that BPD has the potential to be used as a novel sustainable polymeric dye for the high-performance dyeing of organically tanned chrome-free leather, which is paramount to ensuring and promoting the sustainable development of the leather industry.

## 1. Introduction

The leather industry has a long and artistic history, and the goods made of leather are widely used in people’s daily life goods, such as shoes, bags, and sofa and car seat cushions. To obtain leather products, the raw hides/skins from animals must be treated by numerous processes to remove the useless components and transfer the unstable and perishable collagen fibers into stable and durable leather materials. During these processes, tanning is the most critical step, as it irreversibly changes the structure of hide and skin, endowing them with higher durability, lower susceptibility to chemical and microbial decompositions, and better dyeability.

Currently, chromic salts are still widely used in leather tanning because they have a competitive price and can provide excellent physical and organoleptic properties for leather products [1,2]. However, chrome-based tannage will inevitably generate massive chromium-containing leather waste (CCLW), with Cr(III) possibly being converted to Cr(VI) in the manufacturing or storage of leather under some specific conditions [3,4]. Worryingly, Cr(VI) is a well-known hazardous substance due to its toxicity, carcinogenicity, and mutagenic effects on aquatic organisms and humans [5,6]. In addition, the improper disposal of CCLW will cause severe pollution to the soil and groundwater [7]. Although many measures can be taken to tackle these problems, i.e., strict supervision and advanced treatment technologies of CCLW, the environmental regulations are still getting more arduous, and the tanneries are facing severe survival challenges. Therefore, the development and utilization of chrome-free tanning agents (CFTAs) are becoming increasingly urgent for the leather industry [8,9,10].

Among the emerging CFTAs, biomass-derived aldehyde tanning agents (BATs), mainly prepared from saccharide-based biomass via periodate oxidation of the structural unit containing vicinal diols, have recently attracted much attention [11,12]. Such an interest is accounted for by its sustainability, low toxicity, biodegradability, and favorable tanning effects [13,14,15,16]. Despite these auspicious features, BATs are generally non-ionic or anionic, and the –NH_2_ on collagen fibers will be consumed by BATs during the tanning process, thereby resulting in a low isoelectric point (IEP) of BAT-tanned leather that will lead to poor uptake and fixation of the conventional anionic post-tanning materials (CAPMs), which are critical for the post dying stage [17,18]. Accordingly, the discoloration of the leather products may occur during use, which lowers their quality grade. Moreover, the released hazardous synthetic leather dyes will cause potential hazards to human health [19,20]. Given this scenario, exploring environmentally friendly and high-performance dyeing technology for BAT-tanned leather is a promising way to manufacture high-quality chrome-free leather [21,22].

Polymeric dyes are colored polymers that are usually composed of chromophoric groups bonded to the backbone or side chain of the polymer via specific chemical reactions [23]. Since they have adjustable molecular weight, water solubility, and sound absorption capabilities [24], polymeric dyes are excellent candidates to be employed in the high-performance dyeing of leather in the conventional processing system. As previously reported, a kind of waterborne polyurethane-based polymeric dye containing terminal –NH_2_ (AWPUD) could provide higher dyeing uniformity, dry–wet rubbing resistance, and thermal stability for BAT-tanned crust leathers in comparison with conventional small-molecule leather dyes [19]. Meanwhile, the crust leathers prepared from AWPUD dyeing also showed improved physical properties. Conventionally, the backbones of polymeric dyes are synthetic substances derived from petrochemicals, which are chemically very stable and difficult to degrade in a natural environment, which hampers the cost-effectively recycling of polymeric dyes [25,26]. Their extensive usage, accumulation, and soil and water contamination severely threaten the ecosphere, accompanied by increasing carbon footprints and contributing to global warming. Thus, developing polymeric dyes based on renewable and biodegradable resources is beneficial to construct a new post-tanning process for high-quality eco-leather production. In this regard, polymeric dialdehyde polysaccharides (DAP) derived from renewable resources are excellent candidates for developing bio-based polymeric dyes (BPDs) that can be used in the leather industry with excellent quality results [27,28]. Such an extraordinary performance of BPDs is attributed to the presence of reactive –CHO able to react with the small-molecule compounds containing amino groups in the leather. Compared with conventional fossil-based polymeric dyes, BPDs may exert better sustainability and biodegradability because their backbones are made up of sustainable and degradable biomacromolecules, which are significant for constructing an eco-friendlier leather-manufacturing system. Currently, few works have been conducted to create the BPDs that can be used for leather dyeing in aqueous medium.

Given the findings mentioned above and the hypotheses, in this work, dialdehyde starch (DST) and reactive red 180 (RD-180)-containing amino groups were employed to prepare BPDs. The structure features of BPDs prepared with different molar ratios of DST and RD-180 were analyzed by using Fourier-transform infrared spectroscopy (FTIR), nuclear magnetic resonance (NMR), X-ray photoelectron spectroscopy (XPS), gel permeation chromatography (GPC), and UV-Vis spectroscopy. Next, the as-prepared BPD was employed in the dyeing of BAT-tanned leather. The results of this strategy were assessed in terms of the coloring uniformity, color fastness, mechanical strengths, and organoleptic properties of crust leathers. This work represents a novel investigation into developing eco-friendlier polymeric dyes based on sustainable DAP for realizing the production of high-performance eco-leather products that contributes to the greener and cleaner development of the leather industry.

## 2. Materials and Methods

### 2.1. Materials

Dialdehyde starch was provided by Weng Jiang Reagent (Shaoguan, China), and its aldehyde group content was 11.4 mmol/g. Reactive red 180 (RD-180) was obtained from Shanghai Macklin Biochemical Technology Co., Ltd. (Shanghai, China). Absolute ethyl alcohol was of analytical grade and supplied by Tianjin Zhiyuan Chemical Reagent Co., Ltd. (Tianjin, China). A biomass-derived aldehyde tanning agent (BAT, 40 wt%) was prepared using a previously reported method [29]. The –CHO content of BAT was 12.0 mmol/g (based on the absolute dry weight). Conventional anionic dye (CAD) was a commercial product purchased from Jiangsu Aosheng Enterprise Development Co., Ltd. (Gaoyou, China). Pickled sheepskin was purchased from Xinji Lingjue Leather Co., Ltd. (Xinji, China). Other chemicals used in leather processing were of industrial grade and supplied by Sichuan Tingjiang New Material, Inc. (Shifang, China) and Sichuan Dowell Science and Technology Inc. (Chengdu, China).

### 2.2. BAT Tanning of Pickled Sheepskin

Firstly, the pickled sheep skin was weighed. Next, 6% of sodium chloride (based on twice the weight of pickled sheep skin, the same below), 100% of water, and 2% of BAT were added to a drum, with a subsequent running for 5 min. Then, the pickled sheep skin was added to the drum, followed by continuously running for 4 h at room temperature. Afterward, the tanning liquor was alkalized to pH 8.0 through the addition of NaHCO_3_ several times at an interval of 15 min. Subsequently, the temperature of tanning liquor was increased to 40 °C, and the drum kept running for another 4.0 h. After that, the drum stood overnight for sufficient crosslinking between BAT and collagen fibers. After washing with 400 wt% of water at room temperature for 10 min and subsequently horsing up for 24 h, the BAT-tanned leather was obtained.

### 2.3. Preparation of BPD

A certain amount of RD-180 was first dissolved in 250 mL of deionized water, and then 10.0 g of DST was added to the solution. After that, the pH of this aqueous mixture was adjusted to 8.0 by using a sodium carbonate solution (10 g/L), followed by continuously running at 40 °C for 5 h. Next, 500 mL of absolute ethanol was added to precipitate the reaction product at room temperature. After standing for 12 h, the mixture was filtered and separated via vacuum filtration. The obtained filter cake was washed with 200 mL of absolute ethyl alcohol, and then it was lyophilized to obtain BPD, using a vacuum freeze dryer (LC-10N-80A, Lichen Technology, Shanghai, China). When the molar ratio of the aldehyde group of DST to the amino group of RD-180 was 0.5:1, the resultant product was labeled as BPD-1. When the ratio was 1:1, the resultant product was labeled as BPD-2.

### 2.4. Dyeing Trials for the BAT-Tanned Leather

The BAT-tanned leather was shaved to a thickness of 1.0 mm and then weighed. Afterward, the shaved BAT-tanned leather was subjected to the post-tanning process according to the recipe presented in Table 1. After fatliquoring, the processed leather was naturally dried and softened to prepare BAT crust leather, which was subsequently sampled to assess physical properties.

### 2.5. Characterizations

#### 2.5.1. Structural Characterization

FTIR spectra of DST, RD-180, BPD-1, and BPD-2 were recorded using an infrared spectrometer (TENSOR 27, Bruker, Bremen, Germany) via the KBr pressed-disk technique with a resolution of 4 cm^−1^. The ^1^H NMR spectra of these samples were recorded using a nuclear magnetic resonance spectrometer (Avance III 400 MHz, Bruker, Zurich, Switzerland). Each sample was dissolved in D_2_O for the ^1^H NMR measurements. In addition, the elemental compositions and chemical state of RD-180 and BPD-1 were analyzed by XPS (Escalab 250 Xi, Thermo Fisher Scientific Co., Waltham, MA, USA). Gel permeation chromatography (Shimadzu, Rid-20A, Kyoto, Japan) equipped with a TSKgel GMPWXL column (7.8 mm × 300 mm, TOSOH, Tokyo, Japan) was employed to analyze the molecular weight and distribution of these samples. The initial concentration of each sample was 20 g/L, with the injection of eluent (NaNO_3_, 0.1 mol/L, 20 μL). Before injection, the sample solution was filtered through a 0.47 μm pore membrane to eliminate dust particles. The elution temperature was controlled at 35 °C, and the flow rate was set to 0.6 mL/min. Before each test, the test system was calibrated using pullulan standards (Molecular weight: from 6200 to 48,800, Shodex, Tokyo, Japan). The particle sizes of the samples were analyzed using a Zeta potential and nanoparticle size analyzer (Zetasizer, Malvern Panalytical Ltd., Malvern, UK). The UV-Vis spectra of these samples were recorded by ultraviolet spectrophotometer (TU-1810, PERSEE, Beijing, China).

#### 2.5.2. Uptake Ratio of Dye

The retanning/dyeing wastewater was collected to analyze the adsorption of dye. To this end, CAD, RD-180, and BPD starting solutions were prepared and diluted with specific multiples. Next, the absorbance at λ_max_ (540 nm) was tested for calibration. The absorbance of the filtered and diluted wastewater at λ_max_ (540 nm) was detected. The dye uptake ratio was calculated based on Equation (1). The penetration of dye through the cross-section of BAT-tanned leather was recorded using a digital camera.

(1) $$\mathrm{Uptake}\;\mathrm{ratio}\;\mathrm{of}\;\mathrm{dye}\;(\%)=\frac{A_{0\;}{-\;A}_{1}}{A_{0}}\times 100\%\;$$

where A_0_ is the absorbance value of fresh raw solution, and A_1_ is the absorbance value of the wastewater.

#### 2.5.3. Determination of the Physical Property of Crust Leather

Firstly, a colorimeter (SC-80C, Jingyi Kangguang, Beijing, China) was employed to record the color measurement parameters (L^*^, a^*^, b^*^) of eight points from the crust leathers. Then, the total color difference (ΔE) was calculated according to Equation (2).

(2) $$\Delta E=\sqrt{{(\Delta L)}^{2}+(\Delta {a)}^{2}+(\Delta {b)}^{2}}$$

where ∆L is the lightness difference; and Δa and Δb represent the difference of a* and b* values referring to those of a control sample, respectively. The standard deviation (STDEV) value of seven ΔE values was used to assess the dyeing uniformity (control sample: one of the eight points from crust leather). A lower STDEV value means a higher dyeing uniformity [30]. The color saturation was evaluated based on the color parameters and the corresponding STDEV value of crust leather referring to standard white (L^*^ = 95.23, a^*^ = −0.53, b^*^ = 2.06). According to a previously reported method, the dry–wet rubbing fastness of the BAT-tanned crust leather was measured [29].

The resultant crust leathers were air-conditioned for 48 h at 20 °C and 65% Relative Humidity (RH). The mechanical strengths (including tensile strength, tear strength, and elongation at break) of crust leathers were measured using a universal tensile tester (JT7010-A1, Tian Yuan Test Instrument, Yangzhou, China). The softness of the crust leather was tested via the standard GT-303 leather softness tester (Gotech Testing Machines Inc., Dongguan, China), while the method previously reported by Peng et al. [31] was employed to assess the fullness of the BAT-tanned crust leather.

## 3. Results and Discussion

### 3.1. Structural Features of BPD

**Figure 1:**
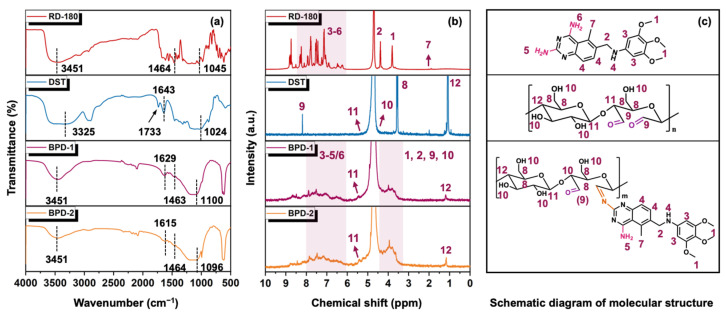
FTIR (**a**) and ^1^H NMR (**b**) spectra and schematic diagram of molecular structures (**c**) of RD-180, DST, and BPD.

First, FTIR and ^1^H NMR were used to characterize the structural features of the as-prepared BPD. Figure 1 illustrates that the RD-180 showed different absorption characteristics: N–H stretching at 3451 cm^−1^, aromatic –C=C– stretching at 1464 cm^−1^, and C–O–C stretching at 1045 cm^−1^ from aromatic nuclei-bonded methoxy groups [32,33]. The DST showed various bands for the following functional groups: 3325 cm^−1^ for –OH stretching, 1733 cm^−1^ for aldehydic –C=O stretching, 1643 cm^−1^ for the scissoring of two O–H bonds of water, and 1024 cm^−1^ for glycosidic bond stretching vibrations [12,34,35,36]. The abovementioned characteristic peaks were preserved in the BPDs with slight shifts, except for the aldehydic –C=O peak. Moreover, the N–H vibration of RD-180 at 3451 cm^−1^ decreased obviously after reacting with DST, indicating that the –NH_2_ groups of RD-180 had reacted with the –CHO groups of DST. These results suggested that most of the aldehyde group of DST was consumed in the reaction between DST and RD-180. These developments also suggested that RD-180 was grafted onto the DST molecule. Figure 1b shows the ^1^H NMR (D_2_O, δ ppm) spectra of RD-180, DST, and BPDs. For RD-180, the typical H from the aromatic nucleus and primary/secondary amino groups lies in 6~8 ppm (3–6), and the signal at 3.81 ppm was assigned to the methoxy group (1). For DST, the ^1^H NMR signal at 8.19 ppm was assigned to the protons of –CH=O [37], which disappeared in the spectrum of BPD. Meanwhile, BPD inherited the other typical signals from RD-180 and DST. These results further confirmed the consumption of aldehyde groups of DST and the reservation of structural features of RD-180 and DST in the BPD. Based on the abovementioned results, it also could be speculated that the Schiff-base structure was formed between RD-180 and DST, thus producing BPD.

**Figure 2:**
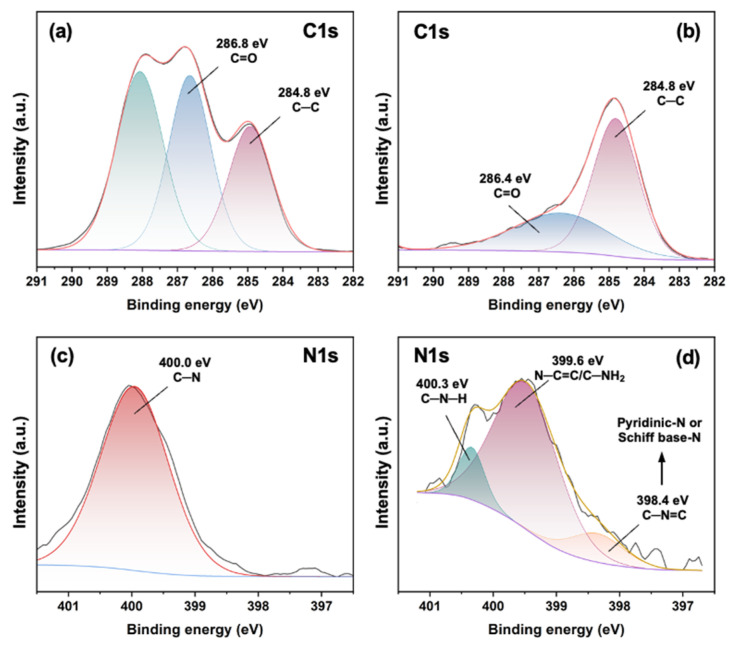
High-resolution C1s XPS spectra of DST (**a**) and BPD-1 (**b**); high-resolution N1s XPS spectra of RD-180 (**c**) and BPD-1 (**d**).

Moreover, the binding states of carbon and nitrogen from RD-180 and BPD-1 were analyzed using XPS. The C1s and N1s high-resolution XPS spectra of RD-180 and BPD-1 are illustrated in Figure 2. 

It can be found from Figure 2a,b that the proportion of the C=O peak (286.8 eV) to C–C peak (284.8 eV) significantly decreased after reacting with RD-180 [38], indicating the incomplete consumption of aldehyde groups of DST. As shown in Figure 2c,d, typically, the spectrum of RD-180 is supposed to have three kinds of N1s signal peaks, which can be overlapped by the C–N (400.0 eV) [39]. Whereas the spectrum of BPD had three kinds of N1s signal peaks, which were attributed to C–N–H (400.3 eV), N–C=C/C–NH_2_ (399.6 eV), and C–N=C (398.4 eV), respectively [39,40,41]. Although the peak of pyridinic nitrogen from RD-180 also appeared around 398 eV [42], in consideration of the increased relative ratio between C–N=C peak and C–N peak, it was suggested the preservation the typical structural feature of RD-180 in the BPD and the formation of more C=N bonds. These results demonstrated the reaction between aldehyde groups of DST and amino groups of RD-180, ensuring that the BPD was successfully prepared.

**Figure 3:**
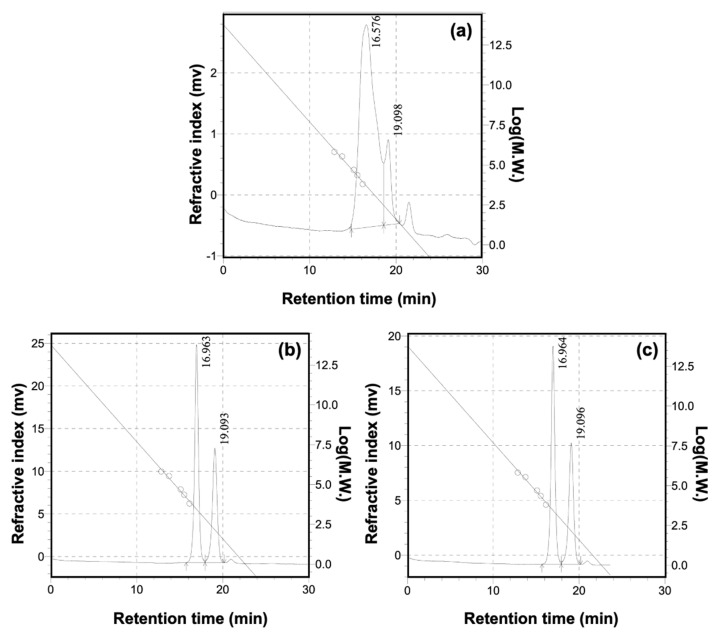
GPC chromatograms of DST (**a**), BPD-1 (**b**), and BPD-2 (**c**).

The molecular sizes of DST, BPD-1, and BPD-2 were analyzed using GPC and nanoparticle size potentiometer. As shown in Figure 3 and Table 2, the weight average molecular weight (M_w_) of DST was 6338 u, while the M_w_ of BPD-1 and BPD-2 decreased to 2997 u and 2990 u, respectively. These diminishments might be due to the cleavage of the backbone of DST during the preparation of BPD at an alkaline condition (pH = 8.0) [43]. Similarly, the particle size of DST decreased from 619 ± 58.3 nm to 359 ± 6.7 nm of BPD. Although the molecular weight was reduced, BPD still showed a polymeric nature.

### 3.2. Color Properties of BPD

**Figure 4:**
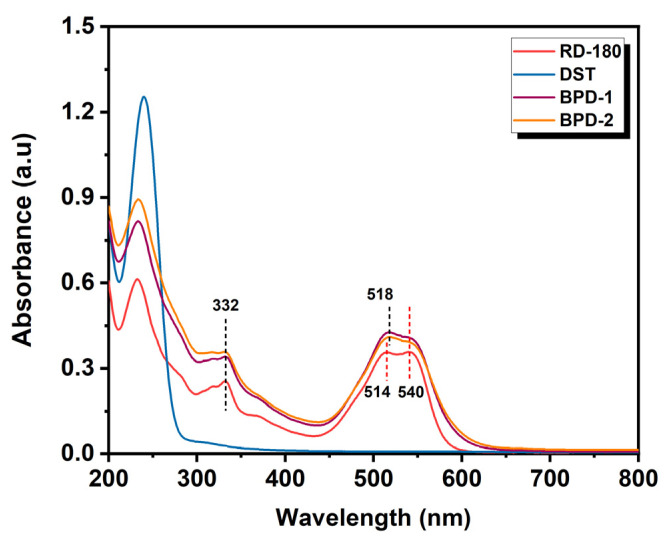
UV-Vis spectra of RD-180, DST, BPD-1, and BPD-2.

The UV-Vis spectra (200–800 nm) of RD-180, DST, and BPDs were recorded and are presented in Figure 4. It can be found that DST had no absorption in the visible range between 400 nm and 800 nm, while RD-180m had two characteristic absorption peaks that appeared at 515 nm and 540 nm, respectively. Figure 4 also reveals that the UV-Vis spectra of BPDs and RD-180 were very similar in terms of the same characteristic absorption peak at 332 nm, with similar characteristic absorption peaks in the visible range. The band in the region of 400–800 nm for RD-180 was ascribed to the N-heterocyclic aromatic rings and benzene rings with substituting groups of the dye molecules in the solution [44]. After being incorporated into the chains of DST, the characteristic peak of RD-180 at 514 nm slightly shifted to 518 nm, showing a bathochromic shift. This might be due to the formation of an extended conjugate system with heteroatomic double-bond groups (C=N) [45] and the polar/polar interaction through hydrogen bonding [46]. Furthermore, based on the UV-Vis spectra data, it could be calculated that the loading degrees (*w*/*w*) of RD-180 on DST-1 and DST-2 were (4.58 ± 0.01)% and (8.73 ± 0.05)%, respectively, after calibration using RD-180 standard solutions. Based on the abovementioned results, it could be demonstrated that RD-180 was successfully loaded onto the chains of DST to produce BPD.

#### 3.2.1. Penetration and Uptake of BPD

**Figure 5:**
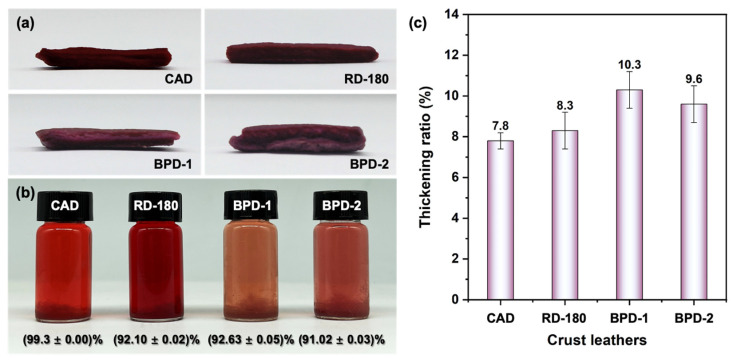
Penetration of different dyes in the leather matrix (**a**), the appearance of filling-dyeing wastewater and the dye’s uptake ratio (**b**), as well as the thickening ratio of crust leather (**c**).

Next, conventional anionic dye (CAD), RD-180, and the as-prepared BPDs were employed in the dyeing of BAT-tanned leather. The dyed-leather was further fatliquored according to the recipe shown in Table 1. After naturally drying, the crust leather was obtained, and the samples were labeled as CAD, RD-180, and BPD crust leather, respectively. Figure 5a illustrates that the macromolecular BPDs had fully penetrated into the BAT-tanned chrome-free leather, exhibiting similar penetrability to CAD and RD-180, which is particularly important for dyeing leather in aqueous media. This development was probably ascribed to the weak electrostatic attractions between the BPD and collagen fiber (CF) matrix resulting from the electronegativity of other fillers and the low IEP of BAT-tanned leather [47]. Figure 5b shows the appearance of filling-dyeing wastewater and the uptake ratio of leather dyes. Compared with the wastewater from CAD dyeing and RD-180 dyeing, the BPD dyeing wastewater had lower chromaticity owing to the lower content of effective coloring components in BPD, as this is beneficial to improving the treatability of comprehensive wastewater. As illustrated in Figure 5c, the thickening ratio of BPD crust leather was higher than that of the CAD crust leather and RD-180 crust leather, suggesting that BPD had a better filling effect than CAD and RD-180.

#### 3.2.2. Dyeing Performance of BPD

**Figure 6:**
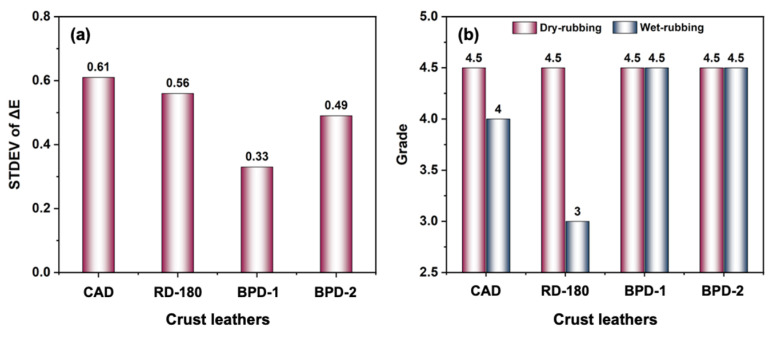
Coloring uniformity (**a**) and fastness (**b**) of crust leathers.

The coloring uniformity and fastness of crust leather were emphatically evaluated. Figure 6a shows the STDEV value of seven ΔE values. As a lower STDEV value means better coloring uniformity [48], the BPD crust leather had higher dyeing uniformity than the CAD and RD-180 crust leathers, thus confirming that BPD displays desirable covering capabilities. The color fastness of crust leather is also paramount to warrant commercial applications besides dyeing uniformity. As shown in Figure 6b, the BPD crust leathers had favorable resistances to dry-rubbing and wet-rubbing (both were 4.5 grade). Although the resistance to dry-rubbing of CAD and RD-180 crust leathers could be up to 4.5 grade, the resistances to wet-rubbing of CAD and RD-180 crust leathers were only 4.0 grade and 3.0 grade, respectively. This suggested that the binding intensity between BPD and the CF matrix was higher than that between CAD/RD-180 and the CF matrix, and the BPD crust leathers thus performed best on resistance to dry/wet-rubbing, while neither CAD nor RD-180 crust leather possessed these two capabilities.

**Figure 7:**
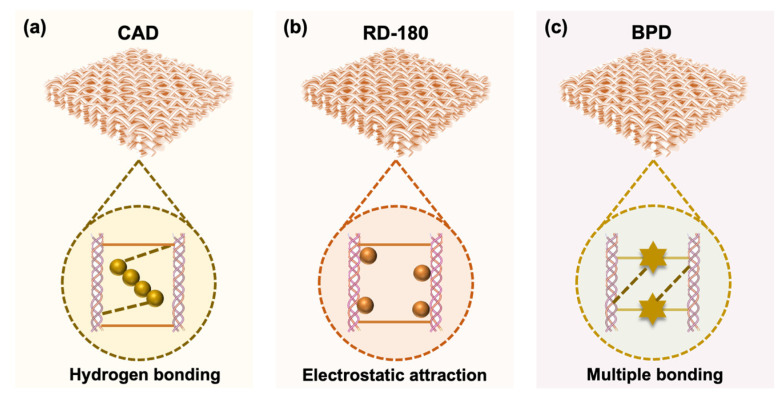
The major ways in which the chromophores were combined in the crust leathers: (**a**) CAD, (**b**) RD-180, and (**c**) BPD.

This difference might be due to the distinct binding ways of the chromophores in the BAT-tanned leather. As illustrated in Figure 7, RD-180 is a small-molecule dye containing amino groups, which can bind with CFs mainly via moderate electrostatic attractions and weak covalent interactions (Figure 7b). CAD is a kind of anionic dye with more oxygen-containing groups and larger molecules than RD-180. Thus, it can interact with CFs via hydrogen bonding and deposit among the CFs caused by the aggregation of molecules by adding formic acid solution in the final stage of the dyeing process (Figure 7a). As a result, it may have stronger binding force than RD-180 in the leather matrix [22]. Although BPD has a larger particle size, it may fully penetrate the interior of BAT-tanned leather at pH 5.5~6.0, benefiting from the entraining effect of CAPMs [49] and then deposit among the CFs caused by the increase of acidity. Meanwhile, BPD might combine with BAT-tanned leather through a slight Schiff base reaction between the –NH_2_ (BPD) and residual –CHO (BAT-tanned leather). Moreover, BPD also has many oxygen-containing groups, which could also be loaded on CFs via forming hydrogen bonds (Figure 7c). Therefore, the as-prepared BPD could be incorporated with the BAT-tanned leather via multiple bonding, including electrostatic interactions and covalent and hydrogen bonds, thus achieving a higher binding intensity of the BPD-treated BAT-tanned leather. This property is significant for realizing high-performance dyeing of organic chrome-free leather.

Table 3 shows the chromatic values and total color difference (ΔE) of the crust leathers. The BPD crust leathers possessed lighter colors than CAD and RD-180 crust leathers given higher ‘L’ and ‘b’ values and lower ‘a’ values owing to the relatively low density of the chromophore groups in BPDs. Therefore, in future research work, BPDs with high chromophore density can be redesigned to give crust leather a highly similar color to CAD crust leather under an exact low dosage to meet commercial application requirements.

#### 3.2.3. Physical and Organoleptic Properties

**Figure 8:**
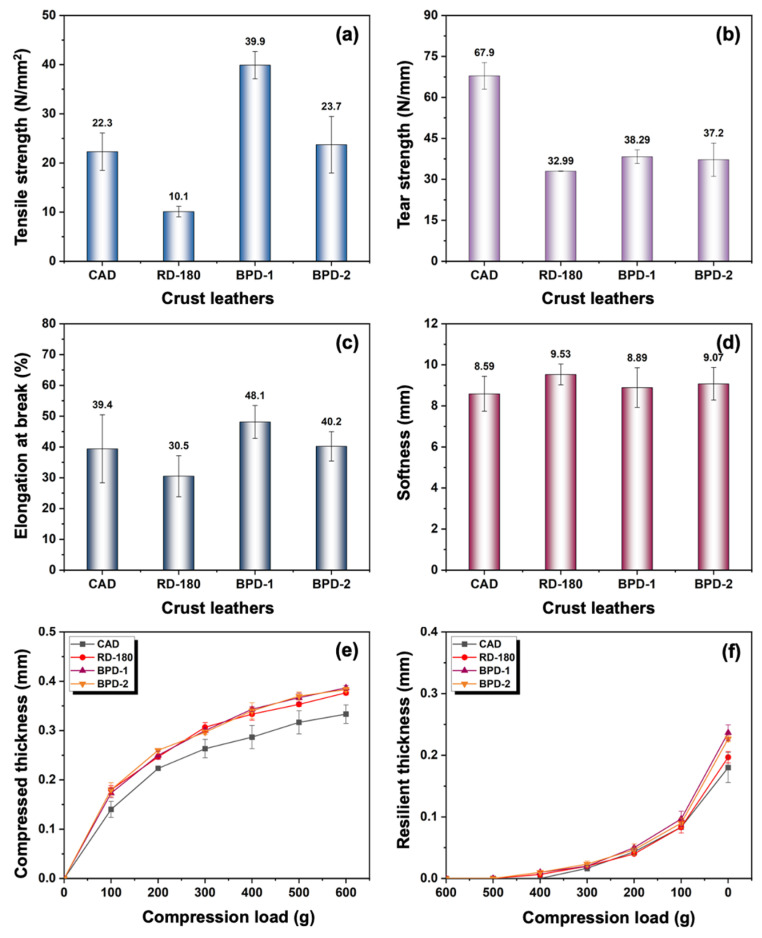
Mechanical and organoleptic performances of crust leathers: (**a**) tensile strength, (**b**) tear strength, (**c**) elongation at break, (**d**) softness, (**e**) compression property, and (**f**) resilience property.

Leather must have favorable physical and organoleptic properties meeting the relevant quality standards to warrant its commercialization. Thus, the physical and organoleptic properties of crust leathers were evaluated. As illustrated in Figure 8a,c, the BPD crust leathers had higher tensile strength and elongation at break than CAD and RD-180 crust leathers. This was attributed to a denser crosslinking CFs network originating from the stronger bindings between dyes and CFs endowed by the –NH_2_ of BPD [19]. However, as shown in Figure 8b, CAD crust leather had the highest tear strength, much higher than RD-180 and BPD crust leathers. This might be due to the relatively excessive binding of RD-180 or BPD species with the CFs that would cause an increase in CF brittleness to lower the tear strength [50]. Figure 8d shows that the softness of RD-180 crust leather was the highest, followed by that of BPD crust leather, with the softness of CAD crust leather being the lowest. This might be ascribed to the relatively higher lubrication degree between CFs resulting from a better uptake of fatliquor caused by the –NH_2_ of RD-180 and BPDs [19]. Generally, higher compressed and resilient thicknesses refer to better fullness of crust leather [51,52,53]. Thus, as shown in Figure 8e,f, the BPD crust leathers performed the best fullness, while CAD crust leather showed the lowest fullness. This might be accounted for by the favorable filling effect of BPDs and the possible higher uptake of other post-tanning materials caused by the–NH_2_ originating from RD-180. In summary, BPD had no significant negative effect on the elongation at break and softness of crust leather, and it could improve the fullness of crust leather. Overall, the BPD crust leathers exhibited satisfactorily comprehensive performances, except for tear strength. Generally, the post-tanning process contains multiple stages. The post-tanning conditions, including the stage of adding BPD, the dosage of BPD, the interactions between BPD, and other post-tanning materials, will affect the performance of BPD in the dyeing of aldehyde-tanned leather. Thus, the post-tanning process can also be adjusted to avoid the embrittlement of CFs and maximize the performance of BPDs in future research work. This would help drive their commercial applications in manufacturing organic chrome-free leathers with high performance to facilitate the sustainable and low-carbon development of the leather industry.

## 4. Conclusions

In this work, a kind of biobased polymeric dye (BPD) was prepared based on dialdehyde starch (DST) and reactive red 180 (RD-180). The FTIR, ^1^H NMR, and XPS analyses suggested that the –CHO groups of DST and the –NH_2_ groups of RD-180 were mostly consumed, revealing the formation of Schiff-base structures between RD-180 and DST. Furthermore, the UV-visible spectrometry demonstrated that RD-180 had been successfully loaded onto the chains of DST, thus ensuring the successful preparation of BPD. The penetrability and uptake capabilities of BPD in the BAT (biomass-derived aldehyde tanning agent)-tanned leather were comparable to that of conventional anionic dye (CAD) and RD-180. Moreover, the thickening ratio of BPD crust leather was higher than that of CAD and RD-180 crust leathers, exerting a better filling effect. Importantly, the BPD crust leather had better dyeing uniformity and color fastness than CAD and RD-180 crust leathers. Compared with CAD, BPD had no significant negative effect on the elongation at break and softness of crust leather, and it could improve the tensile strength and fullness of crust leather. Moreover, the tear strength, elongation at break, and softness of the BPD crust leather were comparable with those of RD-180 crust leather, while the BPD crust leather had a higher tensile strength and better fullness than RD-180 crust leather. Under the experimental conditions, BPDs prepared from different molar ratios between DST and RD-180 had no significant effect on their application performances. In light of these results, this investigation demonstrated that BPDs can be converted to advanced multifunctional polymeric dyes that not only endow the organically tanned chrome-free leather with a high dyeing performance but also result in a possible filling effect, allowing users to dispense with using other filling agents. These excellent features are paramount in developing high-performance eco-leather products, which will contribute a lot to the greener and cleaner development of the leather industry.

## Figures and Tables

**Table 1:** Process formulation of crust leather preparation.

Process	Chemicals	Dosage (%)	Temperature (°C)	Duration/min	Remark
Rewetting	Water	400	40		pH = 6.5
	Degreasing agent	0.5			
	Formic acid	0.5		40	Remark-A
Retanning	Water	100	35		pH = 6.5
	Acrylic resin	3.0		60	
	Amino resin	2.0		30	
	**Dyestuff**	*x*			Remark-C
	Mimosa	4.0		60	
	Formic acid	0.5 × 2		15 × 2	pH = 4.0~4.2
Fatliquoring	Water	150			
	Fatliquor	8.0			
	Formic acid	0.5 × 2		15 × 2 + 20	pH = 3.6~3.8
Washing	Water	400 × 2		10 × 2	Remark-D
Remark-A	Drain				
Remark-B	Drain				
Remark-C	2% for CAD and RD-180, 4% for BPD				
Remark-D	Drain → Natural drying → Softening → Crust leather				

**Table 2:** The molecular weight and particle size of the main components of DST and BPDs.

Samples	M_w_	M_n_	M_w_/M_n_	Particle Size (d.nm)
DST	6338	1869	3.391	619.0 ± 58.3
BPD-1	2997	2658	1.127	452.0 ± 22.2
BPD-2	2990	2641	1.132	359.0 ± 6.7

**Table 3:** Chromatic values and total color difference (ΔE) of crust leathers.

Crust Leather Sample	L	a	b	ΔE
CAD	51.87 ± 0.83	39.37 ± 0.36	14.26 ± 0.36	60.45
RD-180	52.62 ± 0.78	35.47 ± 0.47	0.39 ± 0.31	55.81
BPD-1	64.31 ± 0.69	13.47 ± 0.24	9.05 ± 0.25	34.65
BPD-2	61.51 ± 0.89	17.18 ± 0.40	5.86 ± 0.19	38.28

## Data Availability

All data from this study are presented in the paper.

## Abbreviations

BPD	biobased polymeric dye
DST	dialdehyde starch
RD-180	reactive red 180
BAT	biomass-derived aldehyde tanning agent
CAD	conventional anionic dye
CCLW	chromium-containing leather waste
CFTAs	chrome-free tanning agents
IEP	isoelectric point
CAPMs	conventional anionic post-tanning materials
AWPUD	amino-terminated waterborne polyurethane-based polymeric dye
DAP	dialdehyde polysaccharides
FTIR	Fourier transform infrared spectroscopy
NMR	nuclear magnetic resonance
XPS	X-ray photoelectron spectroscopy
GPC	gel permeation chromatography
ΔE	total color difference
STDEV	standard deviation
CF	collagen fiber
